# Dielectric and Electric Properties of Ba_0.996_La_0.004_Ti_0.999_O_3_ Ceramics Doped with Europium and Hafnium Ions

**DOI:** 10.3390/ma15020413

**Published:** 2022-01-06

**Authors:** Małgorzata Adamczyk-Habrajska, Beata Wodecka-Duś, Tomasz Goryczka, Diana Szalbot, Mateusz Bara, Łukasz Ciepły

**Affiliations:** Faculty of Science and Technology, Institute of Materials Engineering, University of Silesia in Katowice, 41-500 Chorzów, Poland; malgorzata.adamczyk-habrajska@us.edu.pl (M.A.-H.); tomasz.goryczka@us.edu.pl (T.G.); diana.szalbot@us.edu.pl (D.S.); mbara1@us.edu.pl (M.B.); l.cieply@us.edu.pl (Ł.C.)

**Keywords:** ferroelectric properties, ceramics, dielectric properties, impedance spectroscopy

## Abstract

Lanthanum-modified BaTiO_3_ electroceramic materials have superior dielectric and piezoelectric properties. Ba_0.996_La_0.004_Ti_0.999_O_3_ (BLT4) seems to be a serious candidate for ultracondensator applications. This manuscript describes the results of hafnium and europium modification of BLT 4 ceramics. The pure and doped ceramic materials were synthesized by the conventional mixed oxide method. The microstructure of obtained samples was examined by scanning electron microscope. The investigations reveal strong correlations between the presence of admixture and the grain size, which was especially visible in the case of the hafnium dopant. The frequency and temperature dielectric characteristics measurements revealed a decrease in electric permittivity. Moreover, the impedance spectroscopy investigations showed severe changes in grains and grain-boundary resistivity, which was connected with changes in electric conductivity.

## 1. Introduction

Semiconductor ferroelectric ceramics with a perovskite-type structure are an interesting group of materials because of their wide application possibilities [1,2]. These ceramics exhibit piezoelectric, piezoresistive, and posistor properties, which are commonly used in mechatronic systems.

The perovskite structure, with the general formula ABO_3,_ can be represented as a regular type of dense packing. Perovskite materials with this formula (ABO_3_) are characterized by a simple crystal structure: the A position is usually a metal cation from the alkaline or beryllium group (rarely any of the transition metals), B is a cation with a coordination number of six (most often titanium, niobium, tantalum, manganese), and the final part is an oxide anion O^2−^ [3,4]. BaTiO_3_ ceramics are the best-known representatives of this group of materials [5,6]. Although the research history of barium titanate ceramics material is long, investigations have been intensively carried out recently [7,8,9]. BaTiO_3_ ceramics are widely applied, among others, in multilayer ceramic capacitors (MLCCs), positive temperature coefficient thermistor rings (PTCRs), ultrasonic detectors, temperature sensors, dielectric waveguides, multilayer substrates, and microwave integrated circuit substrates [7,8,9,10,11]. The properties of the materials can be easily manipulated by technological conditions as well as admixing with various elements. Substituting homovalent ions (for example Sr^2+^) [12,13,14] or heterovalent ions (for example Sm^3+^, Er^3+^, Ho^3+^, or La^3+^) [15,16,17,18] in the barium position widely changes the observed electric properties. However, one of the best, and most discussed, donor dopants is lanthanum [19,20,21]. Lanthanum barium titanate is a solid solution of barium titanate and lanthanum oxide with the following chemical formula: Ba_1-x_La_x_Ti_1-x/4_O_3_. It should be mentioned that even a small amount of lanthanum (less than 1 at%) significantly changes the properties of barium titanate (BaTiO_3_). A lanthanum admixture induces n-type semiconductivity as well as changes in the polarizability of the material, without significantly altering its crystal lattice. As the stoichiometric formula states, trivalent lanthanum ions are substitutes for subnet A in the perovskite structure, replacing divalent barium ions and disrupting the balance of the electric charges. This equilibrium must be kept by creating cationic vacancies (ion compensation) or by adding an additional electron (electron compensation) [22,23]. This phenomenon can be interpreted through three possible mechanisms: the creation of titanium vacancies, the creation of barium vacancies or electron compensation. Lanthanum-doped barium titanate ceramics are of interest in many research groups. It was observed that increasing the content of lanthanum affects the value of dielectric permittivity and reduces the sintering temperature, which is important from a technological and applicational point of view [24]. In order to choose a proper sintering temperature, it should be noted that above the optimal sintering temperature there are oxygen losses associated with the volatility of this element. In our previous work [25], it was shown that the most favorable concentration of lanthanum was 0.04 mole%. With this amount of dopant, the dielectric permittivity at room temperature was equal to over 40,000, and its maximum value exceeded 100,000 at the phase transition temperature. Ceramics with such excellent dielectric properties are undoubtedly one of the most promising materials for application in ultracapacitors.

Further efforts were focused on searching for modifiers that could improve these dielectric properties. It is well known, that the Eu and Hf dopants used in BaTiO_3_ meet these expectations [26]. For example, the authors noticed that pure BaTiO_3_ is characterized by a low, in terms their application in supercapacitors, dielectric constant (398), while for the Eu modified materials that value increased to 10,576 [27]. Based on data in the literature and our previous results [25], the decision was made to fabricate the Ba_1-x_La_x_Ti_1-x/4_O_3_ material with europium and hafnium ions. However, preliminary measurements clearly showed that the admixtures of hafnium and europium did not meet our expectations. The value of electric permittivity dropped rapidly and there was a significant increase in the loss factor. This fact precluded the use of the discussed materials in ultracapacitors. From the point of view of basic and applicational research, it is crucial to find the reason for drastic and undesirable changes. In the authors’ opinion, the reason for such a state of affairs could be seen in the difference in the resistivity of the grains and grain boundaries. Such a difference is associated with changes in the conductivity paths. Confirmation of this thesis required impedance tests, which are described along with dielectric studies in this article. It is well known that the electrical properties of ceramic materials are closely related to the material’s microstructure. Therefore, the research results presented in this article have been supplemented with microstructural analysis.

## 2. Experiment

Pure Ba_1-x_La_x_Ti_1-x/4_O_3_ and doped ceramics were prepared by the conventional solid-state reaction method. High purity BaCO_3_ (Sigma-Aldrich 99%), La_2_O_3_ (Fluka 99.98%), TiO_2_ (POCH 99.9%), HfO_2_ (Aldrich 98%), and Eu_2_O_3_ (Aldrich 99.5%) were used as raw materials. Stoichiometric amounts of reagents were weighed and mixed in the planetary mill along with ethanol for 24 h until they became homogeneous. The powders were pressed into disc-shaped pellets using a uniaxial hydraulic press. The obtained samples were synthesized at 1223 K for 2 h. After synthesis, the ceramic samples were crushed, further milled, sieved, pressed again into pellets, and prepared for sintering. Those procedures were repeated twice before each sintering. The first sintering was conducted at temperature *T* = 1523 K for 2 h, while the conditions of the second and third sintering process were *T* = 1573 K for 2 h and *T* = 1623 K for 2 h, respectively. The goal of multiple sintering processes was to obtain ceramic materials with well-formed microstructures. The 0.6 mm thick samples were cut, polished, and coated on both sides with silver paste to ensure an appropriate electric contact. After that, the samples with silver electrodes were burned at 923 K for 0.5 h. Morphologies of the BLT4 ceramics were observed with a scanning electron microscope (SEM) (JEOL JSM-7100 TTL LV, JEOL Ltd. Tokyo, Japan). The temperature characteristics of the dielectric loss factor and electric permittivity were measured in a field of several frequencies, selected from the range f = (0.1 ÷ 1000) kHz, in the temperature range *T* = (300 ÷ 800) K. The measurements were performed using a computerized automatic system based on an Agilent E4980A LRC meter (Agilent, Santa Clara, CA, USA). The same system was used in the impedance spectroscopy measurements. The microstructures of the obtained ceramics were examined by a scanning electron microscope and the density was evaluated using the Archimedes method.

## 3. Results and Discussion

**Figure 1:**
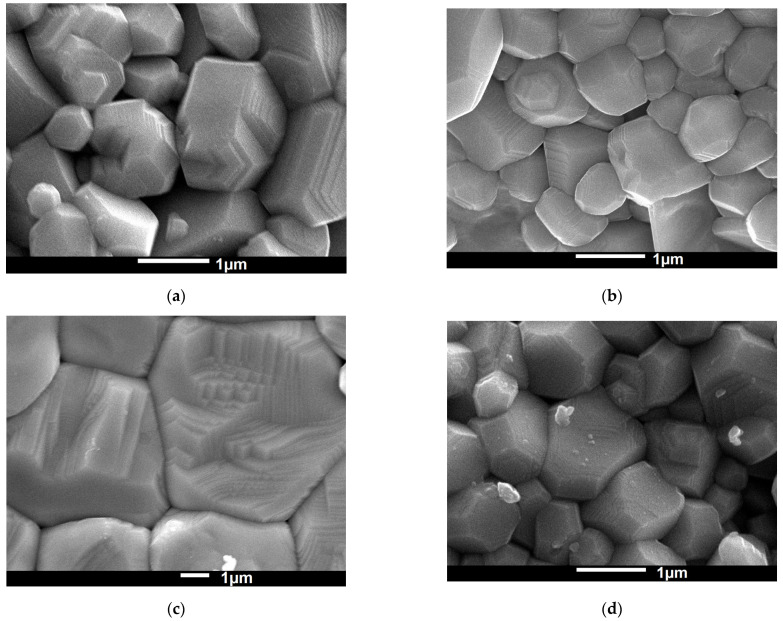
SEM images of the microstructures of the BLT4 ceramics: pure (**a**), doped with 0.4% mol. europium (**b**), 0.4% mol. hafnium (**c**), and 0.4% mol. europium and 0.4% mol. hafnium simultaneously (**d**). Magnification of (**a**,**b**,**d**) is 25,000 and 10,000 in case of (**c**).

The microstructures of the obtained ceramic materials were tested using a scanning electron microscope (Figure 1). The results showed that pure BLT4 ceramics were characterized by well-shaped large grains. The grain’s surface clearly displayed a tendency towards spiraled, hexagonal growth, which favored an increase in single grains and consequently increased the strength of the resulting ceramics [25]. The admixture of europium ions led to significant fragmentation of the grain structure, while the admixture of hafnium increased the average grain size, as well as the packing of the ceramics (Table 1).

The chemical homogeneity of the samples and the absence of impurities were confirmed by the X-ray microanalysis method. Moreover, a quantitative analysis of the chemical composition was carried out. The results for pure ceramics were presented in a previous paper [25]; the results for the hafnium- and europium-modified samples are collected in Table 2, Table 3 and Table 4. All obtained data confirm the high compliance of the actual element content with theoretical stoichiometry. The difference between the theoretical and experimental chemical composition was ±2 wt.%, which was within the error limits of the method used.

**Figure 2:**
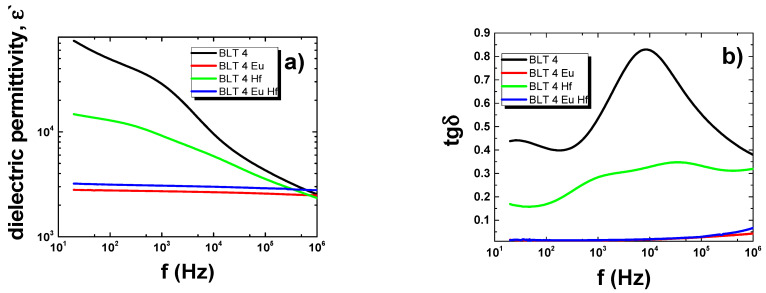
Frequency dependences of dielectric permittivity (**a**) and loss factor (**b**) measured for pure BLT4 ceramics and doped with 0.4% mol. europium, 0.4% mol. hafnium, and simultaneously 0.4% mol. europium and 0.4% mol. hafnium, at room temperature.

The first step in the dielectric measurements of the discussed ceramics was obtaining the frequency characteristics of dielectric permittivity and the loss factor at room temperature (Figure 2). The pure BLT4 ceramics were characterized by extremely high values of dielectric permittivity, especially within the range of low frequency—for a frequency of 100 Hz the value for *ε* was equal to 50,000. The reason for such high values was the appropriate concentration of donor levels and oxygen gaps in the crystal structure of the prepared material [28,29,30,31]. Apparently, the classic solid-phase sintering method provided good conditions for creating oxygen vacancies in the crystal structure of the lanthanum-doped barium titanate ceramics. Unfortunately, the discussed ceramic was also characterized by having a high value for the tangent of the angle of loss, which was equal to 0.41 for a frequency of 100 Hz. The frequency characteristics presented for the modified ceramics clearly indicated a significant deterioration in the dielectric properties of the material. 

**Figure 3:**
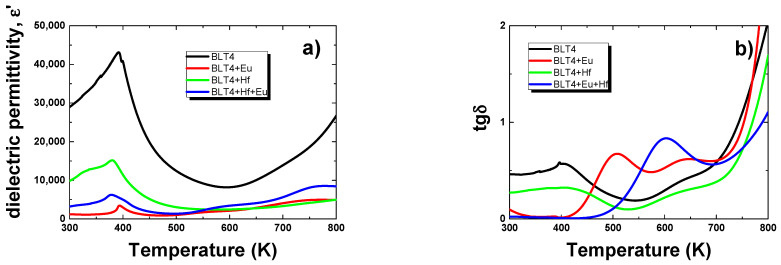
Dielectric permittivity (**a**) and loss tangent (**b**) as a function of temperature, measured at a frequency of 1 kHz, for pure and modified BLT4 ceramics.

The value of dielectric permittivity was significantly reduced, which excluded the applicability of the obtained material, however, the ceramics were very interesting from the point of view of basic research. Due to this fact the decision was made to carry out further research. Figure 3 presents the characteristics of *ε*(*T*) and tgδ(*T*) of pure and modified BLT4 samples at a frequency in the measuring field equal to 1 kHz in a temperature range of 300–800 K.

The maximum that was associated with the transition of the material from the low-temperature ferroelectric phase to the high-temperature paraelectric phase was revealed on all presented characteristics. This maximum took much lower values than in the case of unmodified BLT4 ceramics. Moreover, it shifted towards lower temperatures (Table 5).

In the case of the addition of europium, as well as the addition of both europium and hafnium, the observed maximum of the permittivity had a sharply outlined shape, which indicated the existence of a classic phase transition. However, when it came to the hafnium dopant, the strong broadening of the dielectric permittivity maximum was proof of the existence of a highly diffused phase transition. Shapes of *ε*(*T*) dependencies presented above clearly indicated the ordering action of europium ions introduced into the crystal structure. 

**Figure 4:**
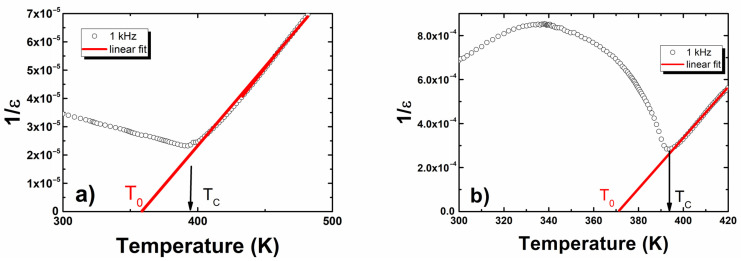
The reciprocal permittivity at 1 kHz as a function of temperature for (**a**) BLT4 and (**b**) BLT4 + Eu ceramics.

In terms of pure BLT4, as well as europium-modified, 1/*ε*(*T*) dependences (Figure 4) satisfied the Curie–Weiss law in a wide range of temperatures in the paraelectric phase, starting from the Curie temperature. These facts indicated that the materials in question underwent a sharp phase transition.

Fitting the experimental data into the Curie–Weiss law allowed us to determine the values of the Curie–Weiss temperature and Curie constant. The values of the obtained parameters are presented in Table 5. In the examined ceramics, the Curie–Weiss temperature was below the Curie temperature, which is a characteristic feature of ferroelectrics with a sharp phase transition.

**Figure 5:**
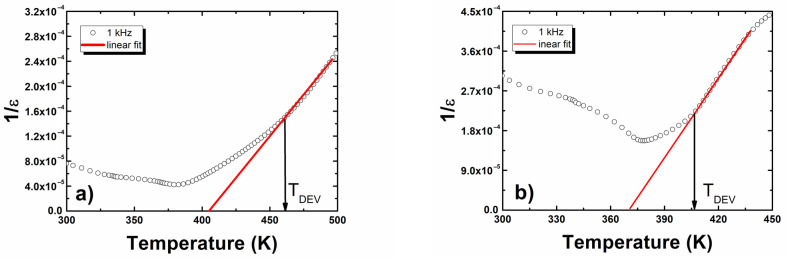
The reciprocal permittivity at 1 kHz as a function of temperature for (**a**) BLT4 + Hf and (**b**) BLT4 + Hf + Eu ceramics.

The 1/*ε*(*T*) dependencies obtained for hafnium-doped BLT4 ceramics, as well as europium- and hafnium-modified BLT4, fulfilled the Curie–Weiss law, which started from temperature *T*_DEV_ (Figure 5).

**Figure 6:**
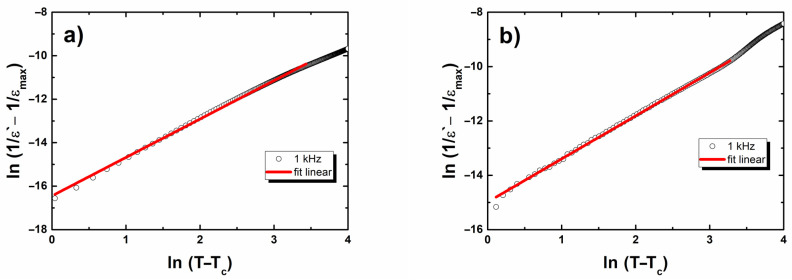
Log(1/*ε* − 1/*ε*_max_) as a function of log(*T* − *T*_*ε*max_) for BLT4 + Hf (**a**) and BLT4 + Hf + Eu (**b**) ceramics. The symbols represent experimental data and the solid line is fit to Equation (1).

Below the *T*_DEV_, the mentioned dependencies are described by modified Curie–Weiss law [32].

(1) $$\frac{1}{\varepsilon ‘}-\frac{1}{\varepsilon {‘}_{\max}}=\frac{{\left(T-T_{m}\right)}^{\gamma }}{C}$$

where *ε_max_* is the maximum value of the dielectric constant at the transition temperature (*T_m_*), *C* is the Curie-like constant, and *γ* is the degree of diffuseness (1 ≤ *γ* ≤ 2). The limiting values *γ* = 1 and *γ* = 2 reduced the expression to the Curie–Weiss law, which was obligatory for the case of a normal ferroelectric and for the quadratic dependence that is valid for an ideal ferroelectric relaxor, respectively. The parameter *γ* was obtained by fitting the experimental data into Equation (1) (Figure 6).

**Figure 7:**
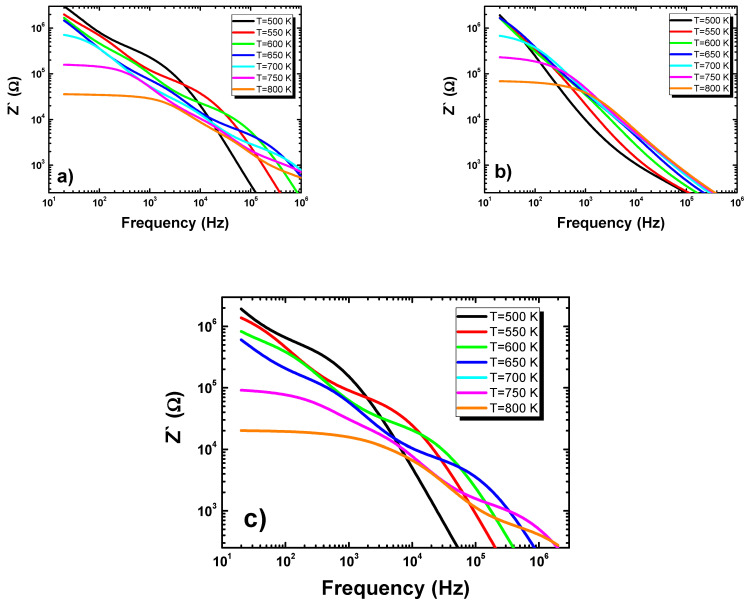
Frequency dependence of the real part of impedance measured at different temperatures for (**a**) europium-modified BLT4 ceramics, (**b**) hafnium-modified BLT4 ceramics, and (**c**) simultaneously hafnium- and europium-modified BLT4 ceramics.

The microstructures of the ceramics are closely related to their electrical behavior. The changes in grain size and material porosity had a strong influence on the conductivity of the materials. The characterization of the electrical behavior of the ceramics was performed by impedance spectroscopy methods. The basis of the technique was the analysis of the AC system response to a sinusoidal perturbation. The analysis led to the calculation of the real (*Z*′) and imaginary parts (*Z*″) of the complex impedance as a frequency function. Figure 7 shows the dependences of the real part of the impedance of frequency at different temperatures and for various admixtures.

In all samples, the magnitude of *Z*′ decreased as the AC conductivity increased. The *Z*′ values obtained at low frequencies (up to 100 Hz) for all discussed ceramics were comparable. The trend gradually changed for higher frequencies, and, consequently, the *Z*′ value above 10^5^ Hz for ceramics modified by Eu was higher than the ones obtained for other discussed materials.

**Figure 8:**
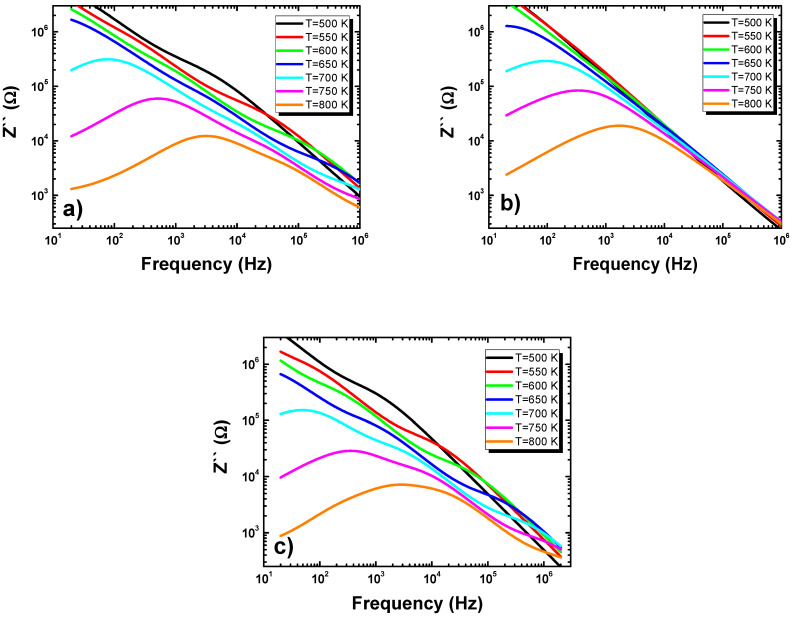
Frequency dependence of the imaginary part of impedance measured at different temperatures for (**a**) europium-modified BLT4 ceramics, (**b**) hafnium-modified BLT4 ceramics, and (**c**) simultaneously hafnium- and europium-modified BLT4 ceramics.

Figure 8 shows the dependences of the imaginary part of the impedance of frequency at different temperatures and for various admixtures. The broadened peaks shifted towards higher frequencies with rising temperatures, indicating the presence of a relaxation process in the system. The electron/immobile species were responsible for the start of the relaxation process at low-temperature regions, whereas the defects were in charge of the process at higher temperatures.

**Figure 9:**
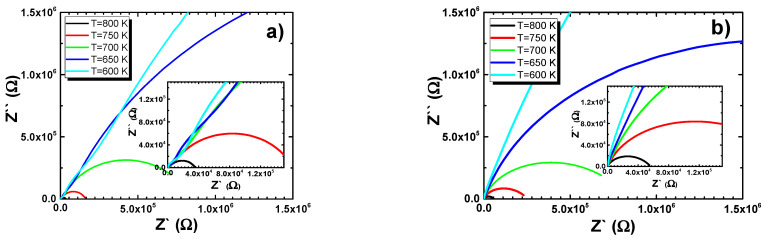

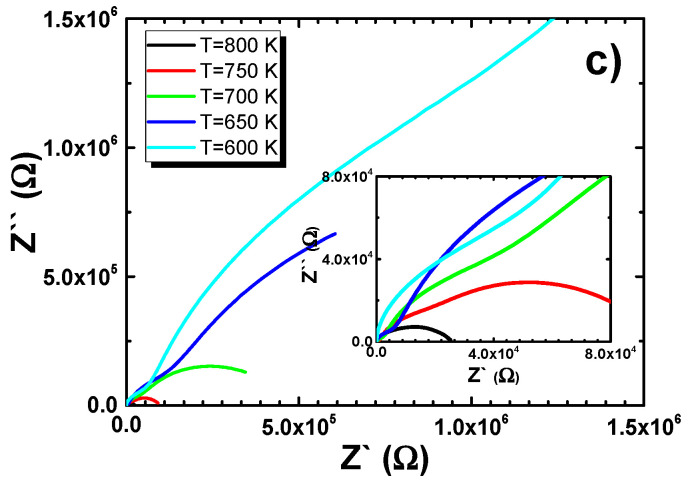
Complex-plane impedance (Nyquist plot) of (**a**) europium-modified BLT4 ceramics, (**b**) hafnium-modified BLT4 ceramics, and (**c**) simultaneously hafnium- and europium-modified BLT4 ceramics.

The next step in the analysis of the discussed results was plotting Nyquist dependences, i.e., the imaginary part of the impedance (*Z*″) as a function of the real one (*Z*′) (Figure 9). The Nyquist plot was a handy tool for examining the electric response of the ceramic materials. 

**Figure 10:**
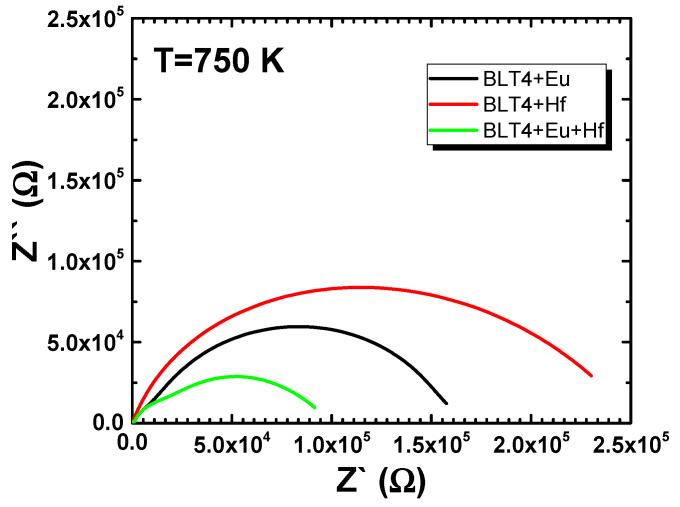
Complex-plane impedance (Nyquist plot) of modified BLT4 ceramics measured at temperature 750 K.

All dependences presented in Figure 9 had the shape of deformed semicircles—the observed deformations resulted from the overlap of two semicircular arcs, which had centers below the axis of the real impedance part. The high-frequency semicircle referred to the bulk (grain) properties of the materials. The second semicircular arc appeared in the low-frequency range of the impedance spectrum and was connected to the grain boundary. Such assignment was consistent with the “brick–layer” model for polycrystalline materials. The degree of deformation was extreme as far as the hafnium-modified BLT4 ceramics were concerned (Figure 10).

**Figure 11:**
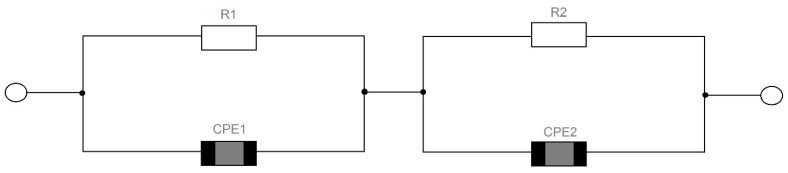
Equivalent circuit used to represent the electrical properties of pure and modified BLT4 ceramics.

It is commonly known that the grain interior and grain boundaries are usually characterized by different values of resistance and capacitance. They can be represented by an equivalent electric circuit consisting of a parallel combination of two resistance and capacitance (RC) circuits connected in series. The values of RC parameters were evaluated from the fitting of the impedance spectrum; however, the quality of the matching was not satisfactory. This was reflected in the high values of the chi-square test χ^2^ (~10^−3^). The equivalent circuit underwent a modification, which was used by many authors, in the compounds based on BaTiO_3_ [24,33], and for other ceramics materials, in which the crystalline structure was significantly different from classical perovskites [34,35]. Namely, two capacitors in the RC circuits were replaced with constant phase elements (CPE1 and CPE2) (Figure 11). The impedance of a CPE element is given as:

*Z*^∗^ _CPE_ = [*A*(*j*ω)*^n^*] ^−1^ (2)

where *A* is a frequency-independent constant and *n* is an exponential index which is a measure of arc depression. For ideal “Debye”-like behavior, the constant *n* = 1 and CPE represented an ideal capacitor with a value *C = A*. The *n* value below unity indicated that a capacitor was frequency dependent. For *n* = 0, the CPE acted as a pure resistor with a value of *R =* 1/*A* [36,37].

**Figure 12:**
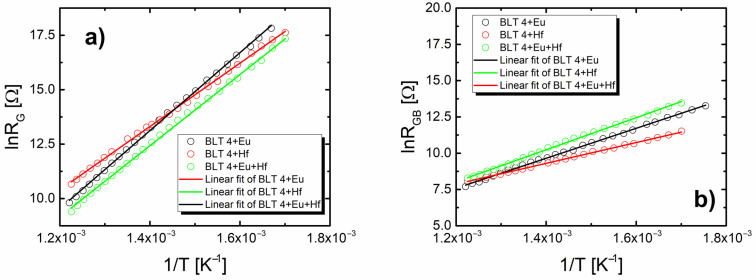
Dependence of the natural logarithm of the grains resistivity (*R*_G_) (**a**) and grain boundaries resistivity (*R*_GB_) (**b**) values calculated on the basis of impedance spectra as a function of the temperature inverse of pure and modified BLT4 ceramics.

According to the author of [38], the need to introduce the changes was a consequence of the reaction rate distribution and/or surface roughness. The modification significantly improved the quality of fitting—the value of the chi-square test χ^2^ was revealed in the order of magnitude 10^−5^. As a result of the adjustment that was made, the temperature dependence of the parameters representing the equivalent circuit was determined. Temperature changes in grain resistance and grain boundaries were particularly interesting from the point of view of the conduction mechanism (Figure 12). 

Both values decreased with a temperature rise, suggesting a negative temperature coefficient in the resistance of the material. Moreover, the obtained results indicated that in the case of all discussed materials the resistivity of grain boundaries (*R*_GB_) was much higher than that of the grains (*R*_G_) (Table 6). 

Dependences of the natural logarithm of the *R*_G_ and *R*_GB_ values versus reciprocal temperature had linear character, which pointed at the activation nature of the conductivity process and could be described by the Arrhenius formula:

*R* = *R*_0_exp(−*E_a_*/*kT*) (3)

Based on this equation, the activation energy of the grain and grain boundary were estimated. Obtained values are collected in the table and compared with the ones received for undoped BLT4 ceramics. In the case of pure ceramics, the values of both discussed activation energies were comparable, which indicated that there was no reduction in ion mobility within the grain boundaries. All the considered modifications of the base ceramics led to a significant increase in the conductivity activation energy within the grain. The activation energy of grain boundaries remains practically unchanged (Table 7). This fact allowed us to state that in the modified ceramics at the higher temperatures the current preferred to flow by the grain boundaries.

## 4. Conclusions

Microstructure analysis confirmed the appropriate selection of sintering conditions. All discussed ceramic materials were characterized by well-formed, angular, and homogenous grains. The grain growth was particularly good for hafnium-doped BLT4 ceramics. The grains were well packed, which resulted in increased density and reduced porosity of the materials. However, the improvement of the microstructure did not connect with the enhancement of the dielectric properties. The value of dielectric permittivity significantly decreased in the discussed samples. For pure ceramics at room temperature, it was equal to 30,000, while in the samples with the addition of hafnium ions it decreased to about 10,000. The value of dielectric permittivity of the other samples discussed in this paper was significantly lower. It is commonly known that the electrical conductivity of ceramics is closely related to the relaxation processes occurring in the interior of the ceramic, i.e., in the grains, in the grain boundaries, and in the electrode areas [39]. To determine the effect of admixture on the electrical properties of BLT4 ceramics, impedance spectroscopy was used as a function of temperature, which confirmed the thesis of the complexity of the mechanism of electric charge transport in the tested ceramic materials. Nyquist’s graphs showed a mutual overlap of two semicircles connected with two microstructure components: grains and grain boundaries. The dominant semicircle was associated with the electrical response of the grain boundaries. The resistance of grains in the modified materials increased compared the to pure BLT4 sample. The values of the activation energy of conductivity in grains (E_G_) and in grain boundaries (E_GB_) in pure ceramics were comparable to each other and equal to 0.87 ± 0.02 eV and 0.89 ± 0.01 eV, respectively. In contrast, the modified ceramics revealed a significant difference in the values of E_G_ and E_GB_ (Table 7). The differences pointed to a change in conductivity paths. Namely, in doped samples, the grain boundaries were mainly responsible for electrical conductivity.

## Figures and Tables

**Table 1:** Density and porosity of discussed ceramics.

Sample	Density [g/cm^3^]	Porosity [%]
BLT4	5.63	7.0
BLT4 + Eu	5.89	2.3
BLT4 + Hf	5.96	1.2
BLT4 + Hf + Eu	5.76	4.4

**Table 2:** Theoretical and experimental percentages of BLT4 + Eu ceramic elements.

Element	Marked Content of Oxides from EDS [%]	Theoretical Content of Oxides [%]	Difference of Determined Value in Relation to the Theoretical [%]
BaO	64.5	65.24	0.74
La_2_O_3_	0.4	0.28	0.12
TiO_2_	34.7	34.18	0.52
Eu_2_O_3_	0.4	0.30	0.1

**Table 3:** Theoretical and experimental percentages of BLT4 + Hf ceramic elements.

Element	Marked Content of Oxides from EDS [%]	Theoretical Content of Oxides [%]	Difference of Determined Value in Relation to the Theoretical [%]
BaO	64.2	64.07	0.13
La_2_O_3_	0.3	0.27	0.03
TiO_2_	31.4	32.13	0.73
HfO_2_	4.1	3.53	0.57

**Table 4:** Theoretical and experimental percentages of BLT4 + Eu + Hf ceramic elements.

Element	Marked Content of Oxides from EDS [%]	Theoretical Content of Oxides [%]	Difference of Determined Value in Relation to the Theoretical [%]
BaO	64.4	63.80	0.6
La_2_O_3_	0.4	0.27	0.13
TiO_2_	31.1	32.10	1.00
Eu_2_O_3_	0.4	0.30	0.10
HfO_2_	3.7	3.53	0.17

**Table 5:** Influence of the modificators on the dielectric parameters of BLT4 ceramics: *T_C_*—Curie temperature, *ε*_max_—maximum value of dielectric permittivity at *T_C_*, *C*—Curie constant, *T_CW_*—Curie–Weiss temperature, and *γ*—diffuseness parameter.

Sample	*T_C_*	*ε*_max_	*C*	*T_CW_*	*γ*
BLT4	399	40,916	1.7 × 10^6^	379	Sharp transition
BLT4 + Eu	393	3453	0.89 × 10^5^	356	Sharp transition
BLT4 + Hf	380	15,174	0.39 × 10^6^	100	1.76
BLT4 + Hf + Eu	378	6247	0.17 × 10^6^	370	1.59

**Table 6:** Values of grain resistivity *R*_G_ and grain-boundary resistivity *R*_GB_ of undoped and modified BLT4, obtained at the three exemplary temperatures.

Ceramics	BLT4		BLT4 + Eu		BLT4 + Hf		BLT4 + Eu and Hf	
Temperature [K]	*R*_G_[kΩ]	*R*_GB_[kΩ]	*R*_G_[kΩ]	*R*_GB_[kΩ]	*R*_G_[kΩ]	*R*_GB_[kΩ]	*R*_G_[kΩ]	*R*_GB_[kΩ]
800	0.8	17.8	29.7	3.3	68.9	3.8	21.2	5.0
700	13.1	267.2	806.2	20.1	842.2	12.9	46.1	39.5

**Table 7:** The values of activation energy of the conduction process in the grain (E_G_) and grain boundaries (E_GB_) of pure and modified BLT4 ceramics.

Sample	E_G_[eV]	E_GB_[eV]
BLT4	0.87 ± 0.02	0.89 ± 0.01
BLT4 + Eu	1.54 ± 0.03	0.89 ± 0.01
BLT4 + Hf	1.25 ± 0.01	0.78 ± 0.01
BLT4 + Hf + Eu	1.58 ± 0.02	0.95 ± 0.02

## Data Availability

Not applicable.

## References

[1] Kinoshita K., Yamaji A. (1976). Grain-size effects on dielectric properties in barium titanate ceramics. J. Appl. Phys..

[2] Hiramatsu T., Tamura T., Wada N., Tamura H., Sakabe Y. (2005). Effects of grain boundary on dielectric properties in fine-grained BaTiO_3_ ceramics. Mater. Sci. Eng. B..

[3] Bochenek D. (2010). Magnetic and ferroelectric properties of PbFe_1/2_Nb_1/2_O_3_ synthesized by a solution precipitation method. J. Alloys Compd..

[4] Bochenek D., Surowiak Z. (2009). Influence of admixtures on the properties of biferroic Pb(Fe_0.5_Nb_0.5_)O_3_ ceramics. Phys. Status Solidi A.

[5] Busca G., Buscaglia V., Leoni M., Nanni P. (1994). Solid-State and Surface Spectroscopic Characterization of BaTiO_3_ Fine Powders. Chem. Mater..

[6] Xu Y. (1991). Ferroelectric Materials and Their Applications.

[7] Tang Y.F., Wu C., Wu Z.X., Hu L., Zhang W., Zhao K. (2017). Fabrication and in vitro biological properties of piezoelectric bioceramics for bone regeneration. Sci. Rep..

[8] Yun W.S., Urban J.J., Gu Q., Park H. (2002). Ferroelectric properties of individual barium Titanate nanowires investigated by scanned probe microscopy. Nano Lett..

[9] Dubourdieu C., Bruley J., Arruda T.M., Posadas A., Jordan-Sweet J., Frank M.M., Cartier E., Frank D.J., Kalinin S.V., Demkov A.A. (2013). Switching of ferroelectric polarization in epitaxial BaTiO_3_ films on silicon without a conducting bottom electrode. Nat. Nanotechnol..

[10] Beck H.P., Eiser W., Haberkorn R. (2001). Pitfalls in the synthesis of nanoscaled perovskite type compounds. Part I: Influence of different sol–gel preparation methods and characterization of nanoscaled BaTiO_3_. J. Eur. Ceram. Soc..

[11] Kim E.S., Liang J.G., Wang C., Cho M.Y., Oh J.M., Kim N.Y. (2019). Inter-digital capacitors with aerosol-deposited high-K dielectric layer for highest capacitance value in capacitive super-sensing applications. Sci. Rep..

[12] Arshad M., Du H., Javed M.S., Maqsood A., Ashraf I., Hussain S., Ma W., Ran H. (2020). Fabrication, structure, and frequency-dependent electrical and dielectric properties of Sr-doped BaTiO_3_ ceramics. Ceram. Int..

[13] Patil D.R., Lokare S.A., Devan R.S., Chougule S.S., Kanamadi C.M., Kolekar Y.D., Chougule B.K. (2007). Studies on electrical and dielectric properties of Ba_1−x_Sr_x_TiO_3_. Mater. Chem. Phys..

[14] Bai Y., Han X., Ding K., Qiao L. (2013). Combined effects of diffuse phase transition and microstructure on the electrocaloric effect in Ba_1−x_Sr_x_TiO_3_ ceramics. Appl. Phys. Lett..

[15] Khushbu P., Kumar V., Kumar J. (2017). Effect of co-substitution of Sm^3+^ and Fe^3+^ ions on structural and dielectric properties of BaTiO_3_ ceramics. Alloys. Compd..

[16] Buscaglia M.T., Viviani M., Buscaglia V., Bottino C., Nanni P. (2002). Incorporation of Er^3+^ into BaTiO_3_. J. Am. Ceram. Soc..

[17] Makovec D., Samardžija Z., Drofenik M. (2004). Solid Solubility of Holmium, Yttrium, and Dysprosium in BaTiO_3_. J. Am. Ceram. Soc..

[18] Bobade S.M., Gopalan P., Choi D.-K. (2009). Dielectric Properties of La^3+^ at A Site and Al^3+^ and Ga^3+^ Doped at B Site in BaTiO_3_. Jpn. J. Appl. Phys..

[19] Cai W., Fu C.L., Lin Z.B., Deng X.L., Jiang W.H. (2012). Influence of lanthanum on microstructure and dielectric properties of barium titanate ceramics by solid state reaction. Adv. Mater. Res..

[20] Mangaiyarkkarasi J., Saravanan R., Ismail M.M. (2016). Chemical bonding and charge density distribution analysis of undoped and lanthanum doped barium titanate ceramics. J. Chem. Sci..

[21] Wang Y., Shi S., Dong Q., Xu C., Zhu S., Zhang X., Chow Y., Wang X., Zhang G., Zhu L. (2021). Electrospun lanthanum-doped barium titanate ceramic fibers with excellent dielectric performance. Mater. Charact..

[22] Morrison F.D., Coats A.M., Sinclair D.C., West A.R. (2001). Charge Compensation Mechanisms in La-Doped BaTiO_3_. J. Electroceramics.

[23] Ianculescu A., Mocanu Z.V., Curecheriu L.P., Mitoseriu L., Padurariu L., Trusca R. (2011). Dielectric and Tunability Properties of La-doped BaTiO_3_ ceramics. J. Alloys Compd..

[24] Mancić D., Paunović V., Vijatović M., Stojanović B., Zivković L. (2008). Electrical Characterization and Impedance Response of Lanthanum Doped Barium Titanate Ceramics. Sci. Sinter..

[25] Wodecka-Duś B., Adamczyk-Habrajska M., Goryczka T., Bochenek D. (2020). Chemical and Physical Properties of the BLT4 Ultra Capacitor—A Suitable Material for Ultracapacitors. Materials.

[26] Garbarz-Glos B., Bąk W., Molak A., Kalvane A. (2013). Microstructure, calorimetric and dielectric investigation of hafnium doped barium titanate ceramics. Phase Transit..

[27] Rath M.K., Pradhan G.K., Pandey B., Verma H.C., Roul B.K., Anand S. (2008). Synthesis, characterization and dielectric properties of europium-doped barium titanate nanopowders. Mater. Lett..

[28] Morrison F.D., Sinclair D.C., West A.R. (1999). Electrical and structural characteristics of lanthanum-doped barium titanate ceramics. J. Appl. Phys..

[29] Kuwabara M., Matsuda H., Kurata N., Matsuyama E. (1997). Shift of the Curie point of barium titanate ceramics with sintering temperature. J. Am. Ceram. Soc..

[30] Vijatović Petrović M.M., Bobić J.D., Ramoska T., Banys J., Stojanović B.D. (2011). Electrical properties of lanthanum doped barium titanate ceramics. Mater. Charact..

[31] Devi S., Jha A.K. (2009). Structural, dielectric and ferroelectric properties of tungsten substituted barium titanate ceramics. Asian J. Chem..

[32] Yu Z., Ang C., Guo R., Bhalla A.S. (2002). Ferroelectric-relaxor behavior of Ba(Ti_0.7_Zr_0.3_)O_3_ Ceramics. J. Appl. Phys..

[33] Mancić D., Paunović V., Petrusic Z., Radmanovic M., Zivkovic L. (2009). Application of Impedance Spectroscopy for Electrical Characterization of Ceramics Materials. Electronics.

[34] Kathayat K., Panigrahi A., Pandey A., Kar S. (2012). Characterization of electrical behavior of Ba_5_HoTi_3_V_7_O_30_ ceramic using impedance analysis. Mater. Sci. Appl..

[35] Parida B.N., Das P.R., Padhee R., Choudhary R.N.P. (2012). Synthesis and characterization of a tungsten bronze ferroelectric oxide. Adv. Mater. Lett..

[36] Komornicki S., Radecka M., Rekas M. (2001). Frequency-dependent electrical properties in the system SnO_2_-TiO_2_. J. Mater. Sci..

[37] Biendicho J.J., West A.R. (2012). Impedance characterisation of LiFePO_4_ ceramics. Solid State Ion..

[38] Amar Nath K., Prasad K., Chandra K.P., Kulkarni A.R. (2013). Impedance and a.c. conductivity studies of Ba(Pr_1/2_Nb_1/2_)O_3_ ceramic. Bull. Mater. Sci..

[39] Abrantes J.C.C., Labrincha J.A., Frade J.R. (2000). An alternative representation of impedance spectra of ceramics. Mater. Res. Bull..

